# The Cheapest Antiseptic

**Published:** 1885-02

**Authors:** 


### The Cheapest Antiseptic.

M. Pasteur anticipates that bisulphide of carbon will become the most efficacious of all antiseptics, as it is also the cheapest, costing but the fraction of a penny per pound in large quantity. It is also the best insecticide known, and for this purpose may, perhaps, be useful to preserve woodwork in tropical countries. Some idea of the use it is already put to may be gathered from the fact that over eight million pounds of the substance are used annually to check the ravages of phylloxera. Carbon bisulphide, as first produced, is about as foul smelling a compound as it is possible to find; but it is capable of purification till all offensive odor is removed, and it is sufficiently pure in smell almost to mix with a perfume.

